# LncRNA HOTAIR promotes LPS-induced inflammatory responses by activating the NF-κB pathway

**DOI:** 10.3389/ebm.2025.10766

**Published:** 2026-01-06

**Authors:** Fengqing Zhu, Zexun Mo, Wuzhou Lin, Cheng Sun, Xiaomei Huang, Meifeng Ye, Hua He, Yujun Li, Kangwei Wang, Juan Zhu, Chuwen Lin, Shuquan Wei, Zhike Liang

**Affiliations:** 1 Department of Pulmonary and Critical Care Medicine, The Second Affiliated Hospital, School of Medicine, South China University of Technology, Guangzhou, Guangdong, China; 2 Department of Pathology, Guangzhou Red Cross Hospital of Jinan University, Guangzhou, Guangdong, China; 3 Department of Histology and Embryology, School of Medicine, Shenzhen Campus of Sun Yat-Sen University, Sun Yat-Sen University, Shenzhen, Guangdong, China

**Keywords:** acute lung injury, HOTAIR, inflammation, LncRNA, NF-κB pathway

## Abstract

Acute lung injury (ALI) is a disease with an excessive inflammatory response triggered by activating the NF-κB signaling pathway. Our study aims to investigate the role of the long non-coding RNA HOTAIR in ALI-associated hyperinflammation, providing evidence for HOTAIR as a potential therapeutic target for ALI. Here, we examined the contribution of HOTAIR to LPS-induced lung injury using both A549 cell and murine models. LPS stimulation markedly increased HOTAIR expression in A549 cells, accompanied by reduced cell viability and elevated secretion of pro-inflammatory cytokines, including IL-1β, IL-6, and TNF-α. Overexpression of HOTAIR further amplified NF-κB signaling, as indicated by increased phosphorylation of IκBα and p65 and enhanced nuclear translocation of p65, whereas silencing HOTAIR effectively reversed these effects. *In vivo*, knockdown of HOTAIR significantly mitigated LPS-induced lung injury, reduced inflammatory cytokine production, and suppressed NF-κB activation in mice. Our findings reveal the contribution of HOTAIR to NF-κB–driven inflammatory injury in ALI, offering insight into its regulatory role and informing future exploration of targeted therapeutic approaches.

## Impact statement

Acute lung injury (ALI) is a life-threatening condition driven by uncontrolled inflammation, yet effective therapeutic targets remain limited. This study identifies the long non-coding RNA HOTAIR as a critical regulator of ALI progression by amplifying NF-κB-mediated hyperinflammation. Using both cellular and animal models, we demonstrate that HOTAIR is upregulated upon LPS exposure and exacerbates lung injury by enhancing NF-κB activation, leading to excessive cytokine release (IL-1β, IL-6, and TNF-α) and tissue damage. Importantly, silencing HOTAIR attenuates inflammation and lung injury, suggesting its therapeutic potential. Our work advances the field by: Establishing HOTAIR as a novel pro-inflammatory driver in ALI, expanding its known roles beyond cancer and chronic diseases. Unveiling a direct link between HOTAIR and NF-κB activation in LPS-induced lung injury, providing mechanistic insight. Proposing HOTAIR inhibition as a strategy to mitigate A LI, offering a new avenue for clinical intervention. These findings could reshape ALI treatment paradigms by targeting epigenetic regulators like HOTAIR to suppress harmful inflammation.

## Introduction

Acute lung injury (ALI) represents a life-threatening pulmonary disorder carrying a significant clinical burden due to its high fatality rates [1, 2]. Bacterial pathogens are key drivers of ALI pathogenesis through LPS-mediated activation of the NF-κB cascade, which orchestrates cytokine storms and subsequent pulmonary tissue destruction [3]. The NF-κB transcription factor complex is composed of five distinct subunits, namely NF-κB1 (p105/p50), NF-κB2 (p100/p52), c-Rel, RelA (p65), and RelB [4]. NF-κB signaling operates through two major branches: the canonical pathway, which is rapidly activated by pro-inflammatory stimuli such as LPS and TNF-α and relies primarily on the p65/p50 heterodimer [5]; and the noncanonical pathway, which involves NF-κB–inducing kinase (NIK)–dependent processing of p100 to p52 and regulates slower immune and developmental processes [6].

Emerging evidence has established long non-coding RNAs (lncRNAs) as critical epigenetic regulators in human pathologies, with notable examples such as GAS5, HULC, NKILA, MALAT1, CASC2, SNHG5, LINC01134, HOTAIR, and PINT demonstrating disease-specific regulatory functions [7–16]. As a well-characterized lncRNA, HOTAIR (HOX transcript antisense intergenic RNA) has emerged as a significant epigenetic regulator in cancer and non-malignant disorders, involving positive feedback loops and compensatory harmful regulation mechanisms [17]. In cancer, HOTAIR contributes to aberrant transcription, chronic inflammation, and treatment resistance via NF-κB–dependent pathways [15, 18–23]. In addition, HOTAIR-NF-κB signaling axis was reported to be involved in aggravating the inflammatory environment of osteoarthritis [24], releasing epithelial-mesenchymal transition (EMT), and airway remodeling during smoke-induced COPD development, and promoting neuronal injury [25–27]. Furthermore, previous studies have shown that HOTAIR activates the NF-κB pathway by promoting p65 phosphorylation, thereby enhancing TNF-α secretion in LPS-stimulated cardiomyocytes from septic mice [28]. Recent studies also link HOTAIR to ALI, demonstrating its roles in regulating epithelial cell autophagy and apoptosis [29], as well as promoting aerobic glycolysis and inflammatory factor secretion through interaction with LIN28 [30]. These findings suggest that HOTAIR contributes to LPS-induced lung epithelial damage and inflammation. However, whether HOTAIR modulates NF-κB signaling in ALI *in vivo* and its broader effects on alveolar epithelial proliferation remain unclear.

This study investigated HOTAIR’s role in ALI by employing A549 cells and LPS-injured mice. Our results reveal that HOTAIR drives inflammatory responses via the NF-κB pathway and influences alveolar epithelial proliferation, providing new mechanistic insights and highlighting HOTAIR as a potential therapeutic target in ALI.

## Materials and methods

### Cells

A549 cells were grown in RPMI-1640 medium (#11875093, Gibco) supplemented with 10% FBS and 1% penicillin-streptomycin (#15140122, Gibco) in an incubator with 5% CO2 at 37 °C. To create a model of lung injury inflammation, A549 cells were exposed to 1 μg/mL of LPS (#L2630, Sigma-Aldrich) for 24 h at 37 °C, seeded into 6-well plates at a density of 1 × 10^6^ cells/well 24 h before transfection. Transfection was performed using Lipofectamine 2000 reagent (#1168027, Invitrogen) according to the manufacturer’s instructions. These cells were harvested for subsequent experiments.

A549 cells were divided into four groups: 1) HOTAIR-overexpression (HOTAIR-OE) group with overexpression of HOTAIR using HOTAIR-pcDNA 3.1 (GenePharma, Shanghai, China); 2) si-HOTAIR group with siRNA specifically targeting HOTAIR for knockdown (GenePharma, Shanghai, China); 3) NC group with pcDNA 3.1 empty vector; 4) si-NC group with siRNA negative control. HOTAIR-OE groups were treated with BAY 11-7082 (10 μM, HY-13453) or an equal volume of DMSO (as control) for 24 h.

### Animals

Eight-week-old wild-type C57BL/6 mice (weighing 20–25 g) were maintained at the Sun Yat-sen University Laboratory Animal Center. All animals were cared for, and procedures followed the approved protocols of the Institutional Animal Care and Use Committee of Guangzhou First People’s Hospital (K-2021-141-01). An acute lung injury animal model was established with gradient concentrations of 5 mg/kg, 10 mg/kg, and 20 mg/kg LPS, and 5 mg/kg was chosen to establish the LPS mice model [Supplementary Figure]. Mice were divided into three groups: 1) Ctrl group with PBS treatment in mice with intratracheal injection of 2.5 nmol of siRNA siNC; 2) LPS group with LPS treatment in mice with intratracheal injection of 2.5 nmol of siRNA siNC; 3) si-HOTAIR group with LPS treatment in mice with intratracheal injection of 2.5 nmol of siRNA si-HOTAIR.

After euthanasia, the right middle lobe was ligated and removed for protein extraction, the right lower lobe was collected for RNA analysis, and the right upper lobe was dissected for the lung wet-to-dry ratio measurement. To ensure proper preservation of airway and alveolar architecture, the trachea was then cannulated, and the remaining lung–heart bloc was fixed by intratracheal instillation of 4% paraformaldehyde (PFA). Following inflation fixation, the tissue was immersed in 4% PFA for 24 h, dehydrated, embedded in paraffin, and sectioned at 7 μm.

### BALF collection and cell counting

The BALF samples were collected by flushing the lung tissues with 0.8 mL of pre-cooled PBS solution twice, centrifuged at 400 g for 5 min at 4 °C, and the cell pellets were collected. The cell pellets were resuspended with PBS and then cytospun onto slides and subjected to Diff-Quik staining (#G1540, Solarbio). Observations were performed using an optical microscope (Zeiss), followed by photography.

The neutrophils, macrophages, and total cell count were quantified, and the ratio of neutrophils to macrophages was calculated. The cell supernatant was stored at −80 °C for protein quantification and ELISA determination.

### Cell proliferation assay

Following a 48-h infection period, A549 single-cell suspensions were plated in 96-well formats with an initial seeding density of 2 × 10^3^ cells/well and digested with trypsin after 24-h incubations. Each group of cells had three replicates. Cell proliferation was assessed using the MTS assay (#G3580, Promega) at 0, 24, 48, and 72 h. The OD absorbance at 490 nm was measured using a microplate absorbance reader.

### RNA extraction and RT-qPCR

Total RNA isolation from cellular or tissue specimens was performed with RNA pure Tissue & Cell Kit (#CW0584, Cwbio), followed by reverse transcription of 1 μg RNA template using PrimeScript RT reagent (#R333, Vazyme), incubate at 50 °C for 15 min and then briefly incubate at 85 °C for 5 s. Quantitative reverse transcription PCR (qRT-PCR) analyses were conducted on the QuantStudio platform (Applied Biosystems) using Vazyme SYBR Mix (#Q712), targeting HOTAIR along with key inflammatory mediators (IL-1β, IL-6, TNF-α) and the NF-κB pathway component NFKB1A (primer sequences in Supplementary Material). The amplification data were quantified using the 2^−ΔΔCT^ method, with the housekeeping gene GAPDH as the normalization control.

### ELISA

Protein lysates were prepared from cellular/tissue specimens using RIPA buffer containing phosphatase and protease inhibitors (#1005, #P1081, Beyotime), followed by quantitative protein assessment with the Thermo Scientific Pierce™ BCA assay system (#23225) for precise concentration determination. ELISA assay kit (#H0109c, #H0149c, #H6156, Elabscience) was used to detect the following biomarkers: The absorbance at 450 nm (A450) was recorded using a microplate reader.

### Western blotting

Electrophoretic separation of protein lysates was conducted using SDS-PAGE, followed by transfer onto PVDF membranes (Merck Millipore #ISEQ00010). Membranes underwent blocking in 5% BSA/TBS (1h, RT) with subsequent TBST washes, then were probed overnight at 4 °C with phospho-specific and total antibodies against key NF-κB pathway components: p-IκBα (#340776), IκBα (#R23322), p-p65 (#310013), p65 (#380172) (all 1:1000, Zen-bioscience). Detection was achieved through 2 h incubation with HRP-conjugated secondary antibodies (Thermo Scientific, 1:10,000). The bands were visualized using SuperSignal West Femto (#34094, Thermo Scientific) and quantified with a chemiluminescence system (Bio-Rad) and ImageJ.

### Immunofluorescence

A549 cells were seeded onto coverslips (#801010, NEST Biotechnology), fixed with 4% PFA for 15 min, washed with PBS, and permeabilized with 0.5% Triton X-100 for 10 min. After blocking with 5% goat serum/0.1% Triton X-100/PBS for 1 hour, samples were incubated overnight at 4 °C with rabbit anti-p65 NF-κB (1:50; #710048, Ebioscience). Cells were then incubated with donkey anti-rabbit Alexa Fluor 488 secondary antibody (1:1000; #ab150073, Abcam) for 1 hour and dyed with DAPI (#D9542, Sigma-Aldrich) for 5 min. Imaging was performed by a fluorescence microscope (magnification ×400; Zeiss Axio Observer 7).

### Lung Wet-to-dry ratio

The Lung Wet-to-dry ratio was determined using the right upper lobe. The right upper lung lobe was excised and rinsed with PBS, and wet weight (WW) was then determined using an analytical balance (accuracy: 0.1 mg). Tissues were then dried in a 60 °C oven for 48–72 h until completely dried (defined as <2% weight variation between 24-h intervals) to determine dry weight (DW). The wet-to-dry ratio was calculated as (WW/DW) × 100%.

### H&E staining

Lung specimens underwent standardized histoprocessing with 4% PFA immersion fixation, followed by graded ethanol dehydration series (70%–100%) and paraffin infiltration for optimal structural preservation. Samples were then sectioned into seven μm-thick slices, the slices were deparaffinized, rehydrated, stained with hematoxylin, rinsed, and counterstained with eosin for 1 min. After dehydration and clearing in xylene, slides were mounted with neutral resin. Observation using a light microscope (Zesis) and photographing were performed using a Digital Pathology Slide Scanner (KFBIO).

Histopathological assessment of lung injury was performed using a semi-quantitative scoring system adapted from Gustavo et.al [31]. Lung sections were evaluated independently by two blinded investigators based on the following criteria: (1) alveolar wall thickening, (2) interstitial or intra-alveolar inflammatory cell infiltration, (3) alveolar edema, (4) hemorrhage, and (5) hyaline membrane formation. Each parameter was scored on a scale of 0–4 (0 = absent, 1 = minimal, 2 = mild, 3 = moderate, 4 = severe). The total lung injury score was calculated as the sum of these individual components.

### Statistical analysis

All statistical computations were performed using GraphPad Prism 9.4.0, with quantitative results expressed as mean ± SD. For comparisons between two groups, Student’s t-test was used. For multigroup comparisons, one-way ANOVA was applied, followed by Bonferroni *post hoc* adjustments. A significance threshold of p < 0.05 considered statistically significant for all experimental conditions.

## Results

### LPS inhibits proliferation, promotes inflammation, and HOTAIR expression

**FIGURE 1:**
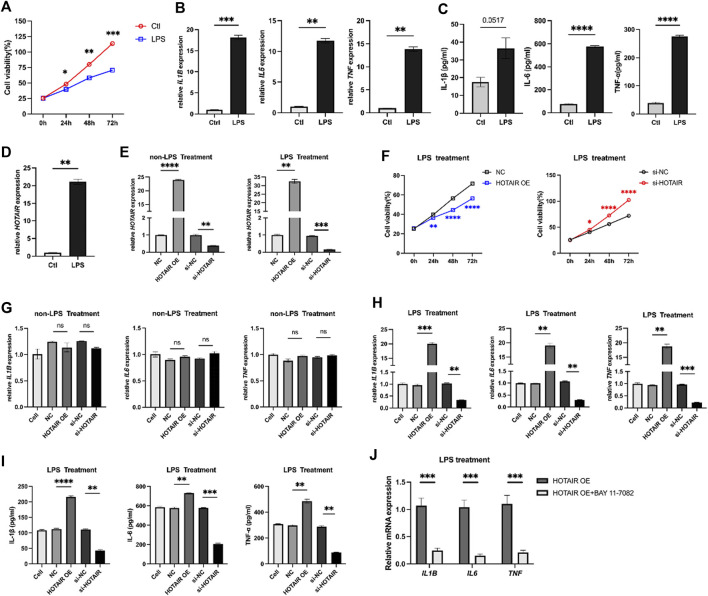
LPS treatment inhibits proliferation, promotes inflammation, and HOTAIR expression in A549 cells. **(A)** MTS assay for cell proliferation in control and LPS treatment A549 cells. **(B)** qPCR for the relative mRNA expression level of *IL1B*, *IL6*, and *TNF* in control and LPS treatment A549 cells, normalized to GAPDH. **(C)** ELISA for inflammatory cytokines, including IL-1β, IL-6, and TNF-α production in control and LPS treatment A549 cells. **(D)** qPCR for the relative mRNA expression level of *HOTAIR* in control and LPS treatment A549 cells, normalized to GAPDH. n = 3. **(E)** qPCR for the relative mRNA expression level of *HOTAIR* in A549 cells after transfection with NC, HOTAIR OE, si-NC, and si-HOTAIR and treatment with/without LPS, normalized to GAPDH. **(F)** MTS assay for cell proliferation in LPS-induced A549 cells after transfection with NC and HOTAIR OE (left), as well as si-NC and si-HOTAIR (right). **(G)** qPCR for the relative mRNA expression level of *IL1B*, *IL6*, and *TNF* in non-LPS treatment A549 cells after transfection with NC, HOTAIR OE, si-NC, and si-HOTAIR, normalized to GAPDH. **(H)** qPCR for the relative mRNA expression level of *IL1B*, *IL6*, and *TNF* in LPS induced treatment A549 cells after transfection with NC, HOTAIR OE, si-NC, and si-HOTAIR, normalized to GAPDH. **(I)** ELISA for inflammatory cytokines production, including IL-1β, IL-6, and TNF-α, in LPS treatment A549 cells after transfection with NC, HOTAIR OE, si-NC, and si-HOTAIR. **(J)** qPCR for the relative mRNA expression level of *IL1B*, *IL6*, and *TNF* in LPS induced treatment A549 cells after transfection with HOTAIR OE, with or with out Bay 11-7082, normalized to GAPDH. n = 3. p < 0.05 (*); p < 0.01 (**); p < 0.001 (***); and p < 0.0001 (****).

A549 cells were treated with LPS to examine its effects on the cells. Cell proliferation was inhibited at 24 h, 48 h, and 72 h after LPS stimulation, compared to the control group (Figure 1A). The levels of mRNA and proteins of pro-inflammatory cytokines IL1B, IL6, and TNF-α were significantly increased in response to LPS stimulation (Figures 1B,C). Furthermore, a notable elevation in the expression of HOTAIR was observed in the group treated with LPS (Figure 1D).

### HOTAIR promotes the inflammatory responses

HOTAIR levels were manipulated in A549 cells, which were then subjected to LPS administration to investigate the role of HOTAIR in ALI. HOTAIR was successfully overexpressed in the HOTAIR OE group, while effective knockdown of HOTAIR was observed in the si-HOTAIR group (Figure 1E). Following LPS treatment, A549 cells overexpressing HOTAIR exhibited significantly lower proliferation levels than the NC group, whereas si-HOTAIR-transfected cells showed significantly higher proliferation than the si-NC group (Figure 1F). Without LPS stimulation, qPCR analysis showed that the mRNA levels of pro-inflammatory cytokines (IL-1β, IL-6, and TNF-α) remained unchanged in both HOTAIR-overexpressing and si-HOTAIR groups (Figure 1G), indicating that HOTAIR alone does not significantly activate NF-κB signaling. Under LPS stimulation, however, HOTAIR-overexpressing cells displayed marked upregulation of IL-1β, IL-6, and TNF-α, whereas these cytokines were significantly downregulated in si-HOTAIR cells at both mRNA and protein levels (Figures 1H,I). Notably, co-treatment with BAY 11-7082 significantly reversed these effects (Figure 1J), reducing the mRNA levels of pro-inflammatory cytokines (IL-1β, IL-6, and TNF-α) comparable to HOTAIR OE group. This suggests that HOTAIR primarily modulates NF-κB–mediated inflammatory responses in the presence of inflammatory stimuli such as LPS.

### HOTAIR activates the NF-κB pathway

**FIGURE 2:**
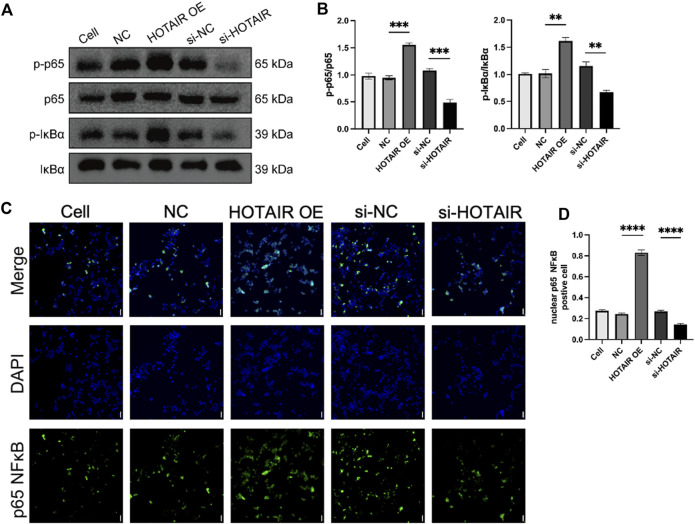
HOTAIR activates the NF-κB pathway by promoting the nuclear translocation of p65 in LPS-induced A549. **(A)** p-IκBα, IκBα, p-p65 NF-κB, and p65 NF-κB protein expression was detected using western blot in LPS treatment A549 cells after transfection with NC, HOTAIR OE, si-NC, and si-HOTAIR. **(B)** Ratio of p-IκBα/IκBα and p-p65 NF-κB and p-p65 NF-κB/NF-κB in LPS treatment A549 cells after transfection with NC, HOTAIR OE, si-NC, and si-HOTAIR. **(C)** Immunofluorescence (IF) for p65 NF-κB in LPS treatment A549 cells after transfection with NC, HOTAIR OE, si-NC, and si-HOTAIR. Scale bar, 50 μm. **(D)** Quantifying nuclear p65 NF-κB positive cells in LPS treatment A549 cells after transfection with NC, HOTAIR OE, si-NC, and si-HOTAIR. n = 3. p < 0.01 (**); p < 0.001 (***); and p < 0.0001 (****).

The NF-κB pathway plays a key role in mediating cell inflammatory response to injury, and HOTAIR has been shown to regulate the NF-κB pathway in osteoarthritis 20. To investigate the molecular mechanisms underlying HOTAIR regulation of inflammation in ALI, we examined whether HOTAIR regulated the NF-κB pathway in this condition. Upon HOTAIR overexpression, the ratios of p-IκBα/IκBα and p-p65/p65 were significantly increased, suggesting activation of the NF-κB pathway (Figures 2A,B). Consistently, upon HOTAIR silencing, the ratios were decreased, suggesting inactivation of the NF-κB pathway (Figures 2A,B). The canonical NF-κB signaling cascade culminates in p65 nuclear localization. Following LPS stimulation, nuclear localization of p65 protein was enhanced in HOTAIR-overexpressing cells, and consistently, it was decreased in HOTAIR-silenced cells (Figures 2C,D). These results suggested that HOTAIR regulates the inflammation in ALI by positively regulating the NF-κB pathway.

### HOTAIR promotes LPS-induced lung injury *in vivo*

**FIGURE 3:**
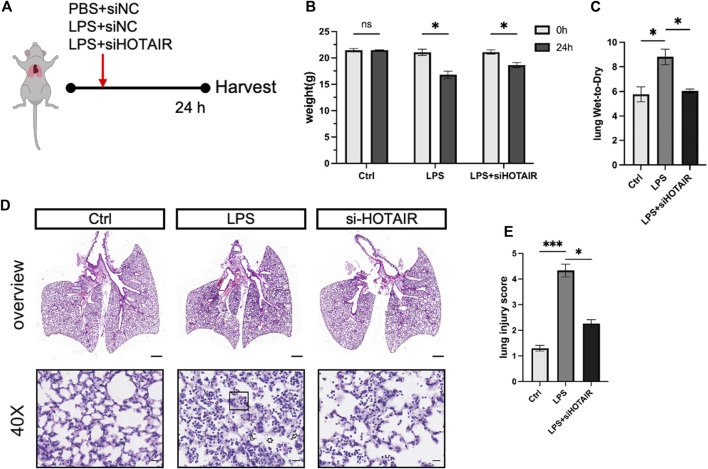
HOTAIR promotes LPS-induced ALI *in vivo*. **(A)** Mice were oropharyngeally administered LPS to simulate ALI, with PBS as the control. si-HOTAIR was co-administered with LPS/PBS simultaneously. After 24 h, mice were euthanized, and lung tissues were harvested. The mice were grouped into PBS + si-NC (Ctrl), LPS + si-NC (LPS), and LPS + si-HOTAIR. **(B)** The body weight (g) at 0 and 24 h of mice in the Ctrl, LPS, and LPS + siHOTAIR groups. **(C)** The lung wet-to-dry ratio (lung W/D) of mice in the Ctrl, LPS, and LPS + siHOTAIR groups. **(D)** Representative histology images of H&E staining from mice in the Ctrl, LPS, and LPS + siHOTAIR groups. Scale bars, 20 μm. Arrowheads: Neutrophils located within the alveolar space or interstitium, indicate alveolar and interstitial inflammation. Box area: alveolar wall thickening and edema. Asterisks: proteinaceous debris filling the airspaces. **(E)** Semi-quantification of lung injury score in the Ctrl, LPS, and LPS + siHOTAIR groups. n = 3 per group; scale bars(black), 50 μm. Ns, not significant; p < 0.05 (*); and p < 0.0001 (****).

LPS treatment significantly upregulated the expression of HOTAIR in a dose-dependent manner, indicating a potential role of HOTAIR in the inflammatory response triggered by LPS (Suppleymentary Figure). To investigate the role of HOTAIR in ALI *in vivo*, we generated HOTAIR-knockdown mice by injection of siRNA si-HOTAIR and stimulated the mice with LPS (Figure 3A). At 24 h post-LPS stimulation, compared to the Ctrl group, the LPS group exhibited significant weight loss and increased lung wet-to-dry ratio (Figure 3B). The si-HOTAIR group also showed weight loss (Figure 3B). On the other hand, the lung wet-to-dry ratio of the si-HOTAIR group was significantly lower than that of the LPS group, and was comparable to that of the Ctrl group (Figure 3C). Histopathological analysis showed minimal lymphocyte and plasma cell infiltration without notable inflammatory response and intact pulmonary architecture in the Ctrl group (Figure 3D, left panel). In contrast, the LPS group exhibited a marked increase in inflammatory cell infiltration, especially neutrophils and macrophages, with localized inflammatory foci; moreover, fluid infiltration was observed in the alveoli, with bronchial wall swelling and obvious interstitial edema, and some alveolar walls became thinner or ruptured, and the integrity of the bronchi and alveoli was compromised (Figure 3D, middle panel). In the si-HOTAIR group, inflammatory cells were mainly concentrated in localized areas with reduced fluid accumulation in the alveolar cavity and mild interstitial edema, and the alveolar wall cells were relatively orderly arranged. The overall degree of inflammation and tissue damage was alleviated compared with the LPS group, but still more pronounced than the control group (Figure 3D, right panel). These results suggested that HOTAIR promotes LPS-induced lung injury *in vivo* (Figures 3D,E).

### HOTAIR promotes LPS-induced inflammation through regulating the NF-κB pathway *in vivo*

**FIGURE 4:**
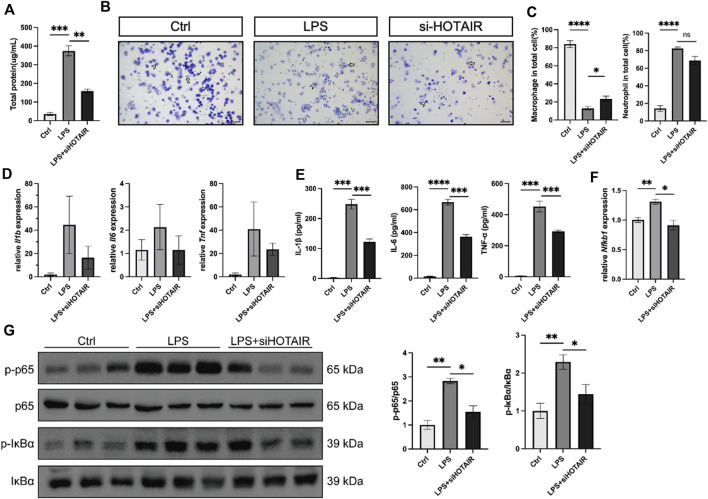
HOTAIR promotes LPS-induced inflammation and promotes LPS-induced inflammation *in vivo*. **(A)** Total protein levels in bronchoalveolar lavage fluid (BALF) from mice after LPS stimulation. n = 3. **(B)** BALF cells from mice in the Ctrl, LPS, and LPS + siHOTAIR groups were cytospun on slides and subjected to Diff-Quik staining. Macrophages and neutrophils were identified based on morphological criteria. Representative images of Diff-Quik staining, Short arrow, macrophage; long arrow, neutrophil. Scale bar, 50 μm. **(C)** Quantification of macrophages and neutrophils as a percentage of total cells in the BALF of mice in the Ctrl, LPS, and LPS + siHOTAIR groups. **(D)** qPCR for the relative mRNA expression level of *Il1b*, *Il6*, and *Tnf* from mice in the Ctrl, LPS, and LPS + siHOTAIR groups, normalized to GAPDH. **(E)** qPCR for the relative mRNA expression level of *Nfkb1* from mice in the Ctrl, LPS, and LPS + siHOTAIR groups, normalized to GAPDH. **(F)** p-IκBα, IκBα, p-p65 NF-κB, and p65 NF-κB protein expression was detected using western blot from mice in the Ctrl, LPS, and LPS + siHOTAIR groups. **(G)** Ratio of p-IκBα/IκBα and p-p65 NF-κB/NF-κB from mice in the Ctrl, LPS, and LPS + siHOTAIR groups. n = 3 per group; p < 0.05 (*); p < 0.01 (**); p < 0.001 (***); and p < 0.0001 (****).

Following LPS stimulation, the total protein levels in bronchoalveolar lavage fluid (BALF) of mice were elevated, which was attenuated in HOTAIR-knockdown mice (Figure 4A). Elevated protein content in BALF reflects enhanced vascular permeability and disruption of the alveolar–capillary barrier, a characteristic pathological feature of ALI. Concurrently, LPS stimulation induced macrophage and neutrophil accumulation in BALF. However, because neutrophils expanded more dramatically, the proportional representation of macrophages appeared reduced. Compared to the LPS group, HOTAIR knockdown showed a mild decrease in macrophage and neutrophil counts, consistent with an attenuated inflammatory response (Figures 4B,C).Further analysis of pro-inflammatory cytokines, including IL-1β, IL-6, and TNF-α, revealed that, while LPS stimulation markedly elevated the protein levels of these cytokines, HOTAIR silencing significantly attenuated this elevation (Figure 4D). Although no statistically significant differences were detected, the mRNA levels of these cytokines exhibited trends similar to protein levels (Figure 4E). Hence, the LPS-induced inflammation was attenuated by HOTAIR silencing.

Next, we investigated the molecular mechanisms by examining the NF-κB signaling pathway *in vivo*. In the mouse lung, LPS stimulation upregulated Nfkb1, a core transcriptional target of the NF-κB pathway, but this upregulation was diminished by HOTAIR silencing (Figure 4F). Furthermore, LPS treatment elevated the ratio of p-p65/p65 and p-IκBα/IκBα, suggesting activation of NF-κB signaling (Figure 4G). Importantly, HOTAIR silencing significantly attenuated the elevation in the ratio of p-p65/p65 and p-IκBα/IκBα, suggesting a compromised NF-κB pathway (Figure 4G). Therefore, LPS-induced activation in the NF-κB pathway was impaired by HOTAIR silencing *in vivo*.

## Discussion

Our study confirmed that HOTAIR activated the NF-κB pathway through key proteins, including p50, p-p65/p65, and p-IκBα/IκBα, in both *in vitro* and *in vivo* models of LPS-induced ALI. This activation leads to the massive recruitment of inflammatory cells, including macrophages and neutrophils, triggers the secretion of pro-inflammatory cytokines, and results in pulmonary tissue damage characterized by inflammatory cell infiltration, tissue edema, and other pathological manifestations.

Serving as the primary driver of inflammatory pathogenesis in LPS-challenged ALI, the NF-κB cascade executes its regulatory dominance through a canonical molecular sequence: IκBα phosphorylation-triggered proteolytic breakdown liberates sequestered p65 subunits for nuclear accumulation, thereby activating transcription of pro-inflammatory mediators [4]. We observed that HOTAIR knockdown could reduce the accumulation of p-IκB and p-p65 induced by LPS stimulation and prevent p65 nuclear translocation, suggesting that HOTAIR knockdown could attenuate severe lung inflammation caused by LPS. Excessive lung inflammation with abnormal macrophage activation is known to be characteristic of ALI. Studies have demonstrated that alveolar macrophages (AMs) increase substantially in BALF following lung injury or inflammation [32, 33]. Interferon drives AM differentiation into M1 macrophages in LPS-induced ALI through recognition receptors such as TLRs, secreting cytokines such as IL-1β, IL-6, IL-18, IL-12, and iNOS, which contribute to the clearance of bacteria and endotoxins, promote the recruitment and infiltration of neutrophils and M1 AM, thereby exacerbating the inflammatory response [34]. Previous studies indicate that HOTAIR regulates NF-κB activation by modulating IκBα degradation in LPS-stimulated macrophages [35]. Whole-transcriptome RNA sequencing analysis identified a series of long non-coding RNAs, including HOTAIR, with potential regulatory functions in cytokine expression and inflammatory responses in macrophages and revealed that HOTAIR exhibits an expression pattern similar to that of pro-inflammatory cytokines following LPS stimulation [36]. In our study, we found that HOTAIR knockdown significantly reduced the expression of macrophages and neutrophils, accompanied by significant reductions in the expression levels of TNF-α, IL-1β, and IL-6, suggesting that HOTAIR knockdown can effectively inhibit the recruitment of macrophages and neutrophils, reducing the expression of pro-inflammatory factors. However, the effect of HOTAIR on macrophage polarization needs to be further confirmed.

ALI is characterized not only by inflammatory cell infiltration but also by epithelial barrier dysfunction. Alveolar type 1 (AT1) cells, a key part of the alveolar-capillary barrier, cover >95% of the gas exchange surface [37]. AT1 cells contain E-NaC, responsible for the bulk of transepithelial Na(+) transport. They may be interfered with under hypoxia or inflammation, triggering fluid retention within the alveolar space and a poor prognosis [38, 39]. Our findings demonstrate that HOTAIR can reverse severe damage to alveolar epithelial cells in LPS-induced ALI in mice, reducing the severity of pulmonary edema, though the direct impact on AT1/AT2 regeneration requires further validation using primary cell cultures or organoid models.

Alveolar type II (AT2) cells exhibit stem-like properties, enabling self-renewal, mobilization, and transdifferentiation into AT1 lineages via an epithelial regeneration program reconstructing alveolar architecture [40]. Our findings demonstrate that HOTAIR knockdown enhances the proliferation of A549 cells in a time-dependent manner, suggesting its potential role in promoting alveolar epithelial regeneration and facilitating lung tissue repair. The main signaling pathways that may promote the proliferation and differentiation of AT2 cells include the Wnt/β-catenin pathway and the YAP/TAZ pathway [41]. The Wnt/β-catenin and YAP/TAZ signaling axes emerge as core regulators of AT2 cell fate determination, orchestrating alveolar regeneration through progenitor cell activation. Notably, HOTAIR exhibits pan-pathological regulatory capacities, driving oncogenic phenotypes in epithelial malignancies (including esophageal/gastric/colorectal carcinomas), modulating vascular calcification in cardiovascular pathologies, governing cartilage homeostasis in degenerative joint diseases, and rewiring placental signaling cascades in hypertensive gestational disorders—predominantly via Wnt/β-catenin-dependent mechanisms [42–48]. Based on these findings, we hypothesize that HOTAIR may mediate the proliferation of AT2 cells in ALI via the Wnt/β-catenin signaling pathway, which requires further confirmation.

Taken together, our findings reveal that HOTAIR amplifies acute inflammatory responses and worsens lung injury largely through NF-κB–dependent mechanisms. HOTAIR silencing mitigated cytokine production, inflammatory cell infiltration, vascular leakage, and tissue destruction, highlighting its potential as a therapeutic target for modulating dysregulated inflammation in ALI. However, it should be noted that the A549 cell system and the single-hit LPS mouse model mainly reflect endotoxin-induced acute injury and do not fully reproduce the clinical heterogeneity of ALI/ARDS, which may arise from bacterial pneumonia, sepsis, aspiration, trauma, or mixed etiologies. Future studies employing clinically relevant models, patient-derived cells, or translational cohorts will be crucial to validate our observations and to further elucidate the therapeutic potential of targeting HOTAIR in diverse ALI/ARDS settings.

## Data Availability

The raw data supporting the conclusions of this article will be made available by the authors, without undue reservation.
