# Supplementary material for: LncRNA HOTAIR promotes LPS-induced inflammatory responses by activating the NF-κB pathway
Source: Exp Biol Med (Maywood). 2026 Jan 6;250:10766. doi: 10.3389/ebm.2025.10766 (PMC12815884; doi:10.3389/ebm.2025.10766)
Supplement: Supplementary file 1 [file DataSheet1.docx]

Table: RT-qPCR primer sequences

| Gene | Target | Forward Sequence(5’-3’) | Reverse Sequence(5’-3’) |
| --- | --- | --- | --- |
| HOTAIR | Human | CAGTGGGGAACTCTGACTCG | GTGCCTGGTGCTCTCTTACC |
| IL-1β | Human | CTTGGTGATGTCTGGTCCAT | CCTTGTACAAAGGACATGGAG |
| IL‐6 | Human | CCAGAGCTGTGCAGATGAGT | CTGCAGCTTCGTCAGCAGGC |
| TNF‐α | Human | CCCACCTTTGCCCGGGGTTC | CCTCCCGGGCGTCAGCACTA |
| GAPDH | Human | GCTCATTTGCAGGGGGGAG | GTTGGTGGTGCAGGAGGCA |
| HOTAIR | Mouse | AGTCCTTCTCCACTTTGCTG | TTGTCTCTAAATCTGGGCACG |
| IL-1β | Mouse | TGGACCTTCCAGGATGAGGACA | GTTCATCTCGGAGCCTGTAGTG |
| IL‐6 | Mouse | TACCACTTCACAAGTCGGAGGC | CTGCAAGTGCATCATCGTTGTTC |
| TNF‐α | Mouse | GGTGCCTATGTCTCAGCCTCTT | GCCATAGAACTGATGAGAGGGAG |
| NFKB1A | Mouse | GCTGCCAAAGAAGGACACGACA | GGCAGGCTATTGCTCATCACAG |
| GAPDH | Mouse | AGGTTGTCTCCTGCGACTTCA | CCAGGAAATGAGCTTGACAAAGTT |

Supplementary figure


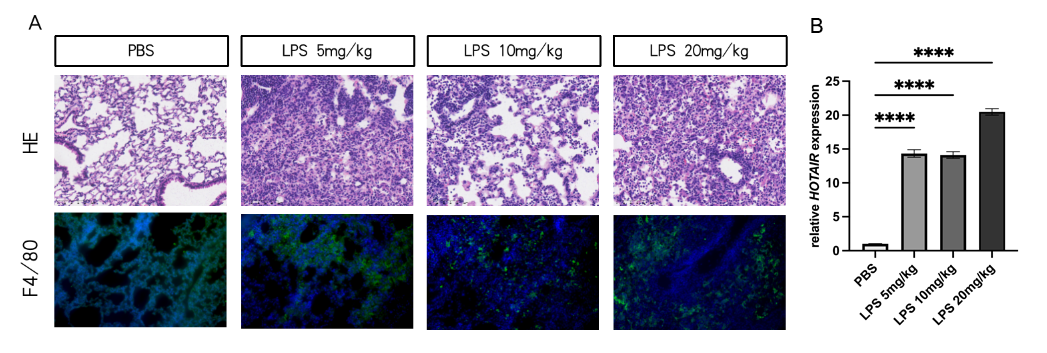


Figure legend:

1. Representative histology images of H&E staining from mice treated with gradient concentrations of LPS. Immunostained for F4/80 from mice treated with gradient concentrations of LPS to assess macrophage infiltration.
2. qPCR for the relative mRNA expression level of *Hotair* in mice lung tissue induced with different doses of LPS, normalized to GAPDH.
